# UCYN-A/haptophyte symbioses dominate N_2_ fixation in the Southern California Current System

**DOI:** 10.1038/s43705-021-00039-7

**Published:** 2021-08-26

**Authors:** Kendra A. Turk-Kubo, Matthew M. Mills, Kevin R. Arrigo, Gert van Dijken, Britt A. Henke, Brittany Stewart, Samuel T. Wilson, Jonathan P. Zehr

**Affiliations:** 1 Ocean Sciences Department, University of California at Santa Cruz, Santa Cruz, CA USA; 2 Earth System Science, Stanford University, Stanford, CA USA; 3 Department of Biological Sciences, University of Southern California, Los Angeles, CA USA; 4 Center for Microbial Oceanography: Research and Education, University of Hawai’i at Manoa, Honolulu, HI USA

**Keywords:** Microbial biooceanography, Biogeochemistry, Water microbiology

## Abstract

The availability of fixed nitrogen (N) is an important factor limiting biological productivity in the oceans. In coastal waters, high dissolved inorganic N concentrations were historically thought to inhibit dinitrogen (N_2_) fixation, however, recent N_2_ fixation measurements and the presence of the N_2_-fixing UCYN-A/haptophyte symbiosis in nearshore waters challenge this paradigm. We characterized the contribution of UCYN-A symbioses to nearshore N_2_ fixation in the Southern California Current System (SCCS) by measuring bulk community and single-cell N_2_ fixation rates, as well as diazotroph community composition and abundance. UCYN-A1 and UCYN-A2 symbioses dominated diazotroph communities throughout the region during upwelling and oceanic seasons. Bulk N_2_ fixation was detected in most surface samples, with rates up to 23.0 ± 3.8 nmol N l^−1^ d^−1^, and was often detected at the deep chlorophyll maximum in the presence of nitrate (>1 µM). UCYN-A2 symbiosis N_2_ fixation rates were higher (151.1 ± 112.7 fmol N cell^−1^ d^−1^) than the UCYN-A1 symbiosis (6.6 ± 8.8 fmol N cell^−1^ d^−1^). N_2_ fixation by the UCYN-A1 symbiosis accounted for a majority of the measured bulk rates at two offshore stations, while the UCYN-A2 symbiosis was an important contributor in three nearshore stations. This report of active UCYN-A symbioses and broad mesoscale distribution patterns establishes UCYN-A symbioses as the dominant diazotrophs in the SCCS, where heterocyst-forming and unicellular cyanobacteria are less prevalent, and provides evidence that the two dominant UCYN-A sublineages are separate ecotypes.

## Introduction

Biological dinitrogen (N_2_) fixation is an important source of new N in N-limited ocean gyres [1]. N_2_ fixation, the energetically expensive process that converts N_2_ into biologically available ammonia, is carried out by diverse Bacteria and Archaea called diazotrophs. N_2_ fixation accounts for up to 70% of new N in the oligotrophic gyres [2], making diazotrophs critical components of open ocean biogeochemical cycles. In contrast, the magnitude and importance of N_2_ fixation in temperate coastal environments is less well understood, having been historically ignored primarily because high concentrations of dissolved inorganic N can inhibit N_2_ fixation [3]. However, there have been numerous recent reports of diazotrophs and N_2_ fixation in various temperate [4–14] and polar [15–17] coastal environments. In one of the most well-studied temperate coastal regions, the Western North Atlantic continental shelf, N_2_ fixation can support up to 50% of net community production [6, 7].

Marine diazotrophic cyanobacteria have diverse morphologies. The unicellular cyanobacterial group A (UCYN-A), which are obligate symbionts of single-celled haptophyte alga related to *Braarudosphaera bigelowii* [18], are emerging as important marine diazotrophs. The basis of the symbiosis is the transfer of photosynthetically-fixed C from the host in exchange for fixed N [18], but other metabolic interdependencies may also be essential [19], since UCYN-A lacks several important metabolic pathways [20, 21]. As such, it differs from the well-studied free-living marine diazotrophs; the biology of the host cell must play a significant role in their biogeography and activity. The impact of the UCYN-A symbiosis on the oceanic N budget is potentially significant due to its broad distribution [22–26], high cell-specific N_2_ fixation and growth rates [27–29], and potential for transfer of fixed N into the food web through grazers [30–32]. UCYN-A symbioses have been reported in regions not typically assumed important for N_2_ fixation, including temperate waters [4, 9, 10, 33], polar seas [16, 17, 26, 34], upwelling and neritic regions [35–37], and the California Current (CC) in the presence of dissolved inorganic N [8, 29]. Despite documentation of UCYN-A symbioses in coastal regions, their N_2_ fixation activity and contribution to the biogeochemistry of these waters is unknown.

This study evaluated N_2_ fixation along nearshore to offshore transects in the Southern California Current System (SCCS), which has been largely ignored as a region with respect to N_2_ fixation, despite net primary production being generally N-limited at the regional scale [38]. Building upon observations of UCYN-A symbioses in the central California Current System [8, 37], this study sought to estimate their contribution to bulk community N_2_ fixation along the continental shelf of the Baja California Sur Peninsula by identifying and quantifying the abundance of different diazotrophs, measuring bulk N_2_ fixation rates (NFRs) and UCYN-A symbiosis single-cell NFRs.

## Materials and methods

### Study location and sample collection

**Fig. 1:**
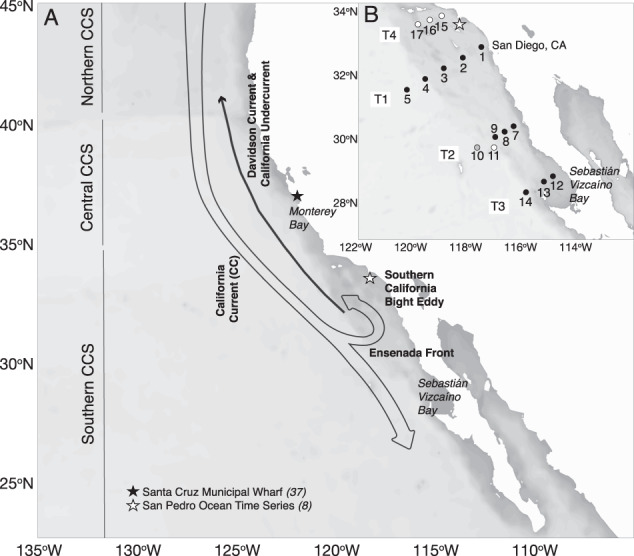
Map of the study region. Schematic of the major currents and regions in the California Current System (**A**). Combined station maps for May 2017 (SP1714) and October 2017 (SP1727) cruises (**B**). Stations sampled during both cruises are solid circles, stations sampled only during SP1714 are gray circles, and stations only sampled in SP1727 are white circles.

Two cruises were conducted aboard the R/V Robert Gordon Sproul from May 3–10, 2017 (SP1714) and October 4–11, 2017 (SP1727) in the waters between San Diego (32^o^ 50.68' N, 117^o^ 31.85' W) and Cedros Island (28^o^ 17.34' N, 118^o^ 12.66' W) in Sebastián Vizcaíno Bay, Baja California Sur, Mexico. Each cruise followed a similar track, with three transects extending from nearshore to offshore waters (Fig. 1). Transect 1 (T1) was along California Cooperative Oceanic Fisheries Investigations (CalCOFI) program line 93, while Transects 2 (T2) and 3 (T3) were along lines 107 and 117, respectively, from the Investigaciones Mexicanas de la Corriente de California (IMECOCAL) program. The October cruise sampled an additional transect north of T1 (T4). Seawater was collected using a rosette of Niskin® bottles equipped with a conductivity, temperature, depth (CTD) package including fluorescence and transmissivity sensors (Seabird, Bellevue, WA), and a photosynthetically active radiation sensor (PAR; Biospherical Instruments, San Diego, CA). At each station, samples from ca. 8 depths between 0 and 200 m were collected for DNA extraction, and dissolved nutrient and chlorophyll *a* (Chl *a*) concentrations. Water for rate incubations were collected from 2 m, 10 m, and the deep chlorophyll maximum.

### Dissolved nutrient and chlorophyll a concentration

Samples for the measurement of nitrate plus nitrite (NO_3_^−^+NO_2_^−^) and phosphate (PO_4_^3−^) concentrations were filtered through precombusted (450 °C for 4 h) 25 mm Whatman^TM^ GF/F filters (MilliporeSigma, Burlington, MA) and stored in acid-cleaned Falcon^TM^ tubes (Thermo Fisher Scientific, Waltham, MA) at −20 °C until analysis using standard techniques [39] on a Lachat QuikChem 8000 Flow Injection Analyzer. The limit of detection (LOD) and limit of quantitation (LOQ), respectively, were 0.01 and 0.04 µmol l^−1^ for NO_3_^−^+NO_2_^−^, 0.01 and 0.02 µmol l^−1^ for PO_4_^3−^. Three separate Chl *a* samples (ca. 150 ml) from each depth were filtered onto GF/F filters, extracted in the dark at 3 °C in 90% acetone for 24 h and measured fluorometrically using a Turner Fluorometer TD-700 (Turner Designs, Inc., San Jose, CA) as described in Welschmeyer et al. [40]. The LOD, calculated as 3× the standard deviation of the blank (90% acetone), was 0.002 µg Chl *a* l^−1^. With the exception of 200 m samples at five stations, the coefficient of variation for the averaged values was <10%.

### DNA collection and extraction

Seawater was sampled into acid-cleaned polycarbonate 2 l bottles and filtered through Sterivex^TM^ (MilliporeSigma) filters using gentle peristaltic pumping. Sterivex^TM^ filters were capped, flash-frozen in liquid N_2_, and stored at −80 °C. DNA was extracted using the DNeasy Plant Kit (Qiagen, Germantown, MD) using modifications to the manufacturer’s guidelines described in detail in Moisander et al. [41]. On-column steps were automated using a QIACube (Qiagen). DNA was quantified using the Picogreen® dsDNA Quantitation kit (Molecular Probes, Eugene, OR).

### Diazotroph community composition and UCYN-A *nifH* oligotyping

Diazotroph community composition was characterized using *nifH* amplicon sequencing using a custom pipeline described in detail by Cabello et al. [37]. Operational taxonomic units (OTUs; herein identified as “denovo”) were defined at 97% nucleotide identity in QIIME [42] using Usearch6.1 [43, 44]. A total of 4,288,726 *nifH* sequences remained after removing low quality sequences and OTUs with <100 sequences, ranging from 1 to 54,726 per sample. The resulting OTU table was subsampled to 2312 sequences, which removed 33 samples primarily from SP1714. Taxonomy was assigned via BLASTX using full length *nifH* amino acid sequences (publicly available at jzehrlab.com/nifh) with *nifH* cluster designations based on Zehr et al. [45]. Raw sequences are available in the NCBI Sequence Read Archive (BioProject PRJNA695866).

UCYN-A *nifH* sequence types were resolved using oligotyping [46], using the exact entropy positions and oligotyping arguments described by Turk-Kubo et al. [47]. To reevaluate global patterns of UCYN-A oligotype distributions, SCCS data were combined with global survey data [47] and other recently published datasets from the Noumea Lagoon in New Caledonia [48], Bering and Chukchi Seas [17], North Pacific Subtropical Gyre (NPSG) [49], and Monterey Bay, CA [37]. This compiled dataset was rarefied to 1029 sequences prior to ordination analyses using Phyloseq [50] in R (r-project.org). The dissimilarity between samples was determined using Bray–Curtis ecological indices and Principal Coordinate Analysis (PCoA) on the resulting distance matrices to visualize dissimilarity between samples and co-occurring oligotypes.

### Targeted diazotroph abundance estimates via qPCR

Gene-based abundance estimates of UCYN-A1 [51], UCYN-A2 [52], *Crocosphaera* (UCYN-B; [24]), *Trichodesmium* [51], *Richelia* associated with *Hemiaulus* (Het-2; [53]), and gamma A (γ-24774A11; [41]) were determined using Taqman® qPCR assays. Protocols used for all aspects of qPCR analysis, including reaction conditions, the use of linearized plasmids and inhibition reactions, and calculation of unknowns follow those described in detail by Goebel et al. [54], apart from a 64 °C annealing temperature for the UCYN-A2 assay. The LOD and LOQ for all assays ranged between 25-31 and 200-250 *nifH* copies l^−1^, respectively. Targets with *nifH* copies >LOD and <LOQ are detected not quantified (DNQ).

The UCYN-A1 qPCR assay has high specificity, but the UCYN-A2 assay cross-reacts with UCYN-A3 and UCYN-A4 [55]. SCCS UCYN-A assemblages were dominated by UCYN-A1 and UCYN-A2; UCYN-A4 was present at low relative abundance, while UCYN-A3 was rarely detected. Therefore, *nifH*-based abundance using the UCYN-A2 assay may also include UCYN-A4.

### Bulk community N_2_ fixation rate measurements

Seawater was sampled directly from Niskin® bottles into acid-washed 1.2 l polycarbonate bottles through 210 µm Nitex® mesh (Wildco, Yulee, FL) to remove large grazers. Incubation bottles received 100 ml of ^15^N_2_-enriched seawater. ^15^N_2_-enriched seawater [56] was generated and atom% enrichment was measured according to procedures described in detail by Mills et al. [29]. The ^15^N_2_-enriched seawater atom% enrichment ranged from 2.0–6.1% for SP1714 and 5.1–24.7% for SP1727. Bottles were incubated (24 h) under simulated in situ light using neutral density screening and maintained at surface seawater temperatures in flow-through on-deck incubators. Samples for atom% ^15^N of the ambient particulate matter were taken from corresponding depths at T_0_. At the termination of the incubation, samples for the analysis of ^15^N enrichment into particulate organic matter (ca. 1000 ml) were processed and measured, and NFRs were calculated, as detailed in Mills et al. [29]. LOD and minimum quantifiable rates (MQRs) were calculated as in Montoya et al. [57] and Gradoville et al. [58] in accordance with recommendations by White et al. [59]. LODs ranged from 0.2–3.0 nmol N l^−1^ d^−1^ (May) and 0.3–5.4 nmol N l^−1^ d^−1^ (October).

### UCYN-A symbioses single-cell N_2_ fixation rates

Subsamples (95 ml) taken from the incubation bottles were fixed with sterile-filtered formaldehyde (MilliporeSigma) at a final concentration of 1.85% (v/v) for >1 h at 4 °C, then concentrated with 0.6 µm pore-size polycarbonate filters (MilliporeSigma) under gentle vacuum, air-dried and stored at −80 °C. UCYN-A1 and UCYN-A2 symbioses were targeted using 5′-horseradish peroxidase-labeled oligonucleotide probes (Biomers, Inc., Ulm/Donau, Germany), using helper and competitor probes for both symbionts and hosts (Biomers) as described in detail by Cornejo-Castillo et al. [60]. Protocols for CARD-FISH hybridizations followed procedures described in detail by Cabello et al. [61].

Samples were visualized, transferred, and mapped to facilitate nanoSIMS analyses according to protocols detailed in Mills et al. [29]. Individual symbioses were analyzed on a Cameca nanoSIMS 50 L at the Stanford Nano Shared Facilities (Stanford, CA). Once targets were located using the charged-coupled device camera and the secondary electron image, image fields were rastered with a 16 keV Cesium primary ion beam (~5 pA) focused into ca. 120 nm spot diameter (256 × 256 pixels, dwell time 1 ms per pixel). Images of ^12^C^−^, ^13^C^−^, ^12^C^14^N^−^ and ^12^C^15^N^−^ were measured over 30–100 planes with a mass resolving power of ca. 8000. Regions of interest were defined around UCYN-A and host cells using Look@nanoSIMS [62]. Isotope ratios of UCYN-A and haptophyte cells were calculated as described in Mills et al. [29]. Single-cell NFRs were determined as follows

$$\ N_{2}\,fixation\,rate\,({\it{fmol}}\,{\it{cell}}^{ - 1}{\it{d}}^{ - 1}) = \frac{{A{}_{\it{PN}}^{\it{final}} - A_{\it{PN}}^{t = 0}}}{{(A_{{\it{N}}_2} - A_{\it{PN}}^{t = 0})}} \ast \frac{{[{\it{PN}}_{\it{cell}}]}}{{\Delta t}}$$

where *ρ* equals the absolute uptake rate per cell, $A\,_{\it{PN}}^{\it{final}}$ and $A\,_{\it{PN}}^{t = 0}$ equal the atom% ^15^N of the enriched (*final*) or unenriched (*t* = 0) UCYN-A symbiosis, $A_{{{{{{\rm{N}}}}}}_2}$ is the atom% enrichment of the N_2_ source pool, Δ*t* is the incubation time, and $\left[ {{\it{PN}}_{\it{cell}}} \right]$ is the per cell N quota. Cell biomass estimates utilized biovolumes according to Krupke et al. [63] and were converted to per cell N quotas using C:N estimates from Martinez-Perez et al. [28]. As with bulk NFR, the LOD and MQR were calculated as in Montoya et al. [57] and Gradoville et al. [58]. NFRs for associated UCYN-A and haptophyte cells were calculated individually and then summed to obtain a single-cell NFR for the entire symbiosis.

Contributions of UCYN-A symbioses to bulk NFR were estimated using single-cell NFRs and *nifH*-based abundance assuming 1 *nifH* copy cell^−1^ for the UCYN-A1 symbiosis and 10 *nifH* copy cell^−1^ for the UCYN-A2 symbiosis, which is at the high end of the estimated *nifH* copies cell^−1^ range previously reported [52]. At present, this is the best estimate that can be justified, but may result in an underestimation of UCYN-A2 symbiosis contribution. Maximum single-cell NFRs were determined using a dilution factor of 75% to correct for isotope dilution during sample processing [64].

### Statistical analysis

Normality was assessed using the Shapiro–Wilk test. The association between diazotroph abundance, NFR, and measured environmental variables was evaluated using the non-parametric Spearman’s rank correlation (*ρ*), to account for the non-normal distribution of some variables. General linear models were also developed (see Supplemental methods). Both approaches agreed overall; Spearman’s *ρ* results are emphasized in the Results and Discussion sections. Comparison of seasonal means for environmental parameters was evaluated using the Mann–Whitney *U* test or t-test for non-normally and normally distributed data, respectively.

## Results

### Seasonal differences in oceanographic conditions

**Fig. 2:**
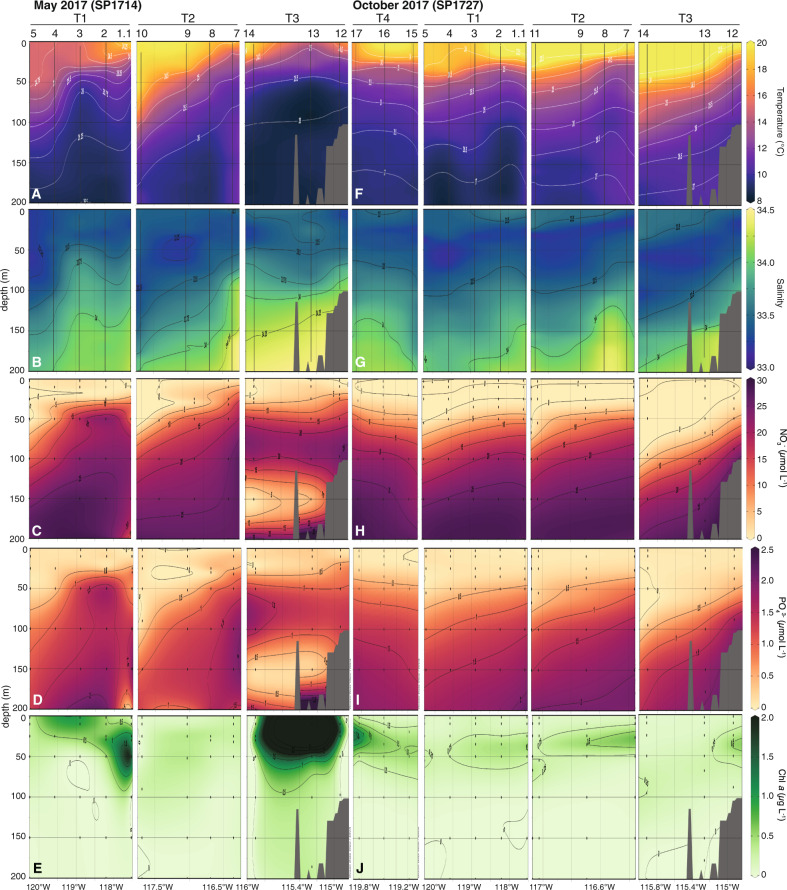
Physical, chemical and biological conditions in the SCCS. Section plots of temperature (**A**, **F**), salinity (**B**, **G**), NO_3_^−^ (**C**, **H**), PO_4_^3−^ (**D**, **I**), and Chl *a* (**E**, **J**) along T1–T4 from May 2017 (SP1714 cruise; **A**–**E**) and October 2017 (SP1727 cruise; **F**–**J**). Transects and stations along each transect are noted at top.

Surface water (0–50 m) temperatures were lower in May (14.4 ± 2.2 °C) than in October (17.5 ± 3.2 °C) (Fig. 2A, F, Tables S1 and S2) while salinity differences were small (0.1 ± 0.2) between expeditions (Fig. 2B, G). The influence of tropical surface water in the study region was evident in October where surface waters had a potential density anomaly (γ_θ_) < 24 kg m^−3^, a signature of poleward transport of tropical surface water [(65]; Fig. S1). During May, the depth of the mixed layer increased with distance from shore (Tables S1 and S2). Along T1 and T2, mixed layer depths averaged 15 ± 17.5 and 18 ± 12.2 m, respectively, with the deepest mixed layers at stations furthest from the coast. Along T3, mixed layer depths were shallower (10 ± 6.7 m), but also deepened offshore. In October, mixed layer depths were shallower and less variable, averaging 11 ± 1.9 m, 10 ± 1.3, and 8 ± 3.9 m along T1, T2, T3, respectively.

The Bakun Upwelling Index [66] indicated upwelling just prior to the May cruise along all transects (Fig. S2). Upwelling decreased by the start of the cruise and remained relatively weak during T2 and T3. A large upwelling event took place on May 7 along T1, which preceded the re-sampling at Stn. 1 (Stn 1.1) and Stns. 2–5. In October there were no distinct upwelling events prior to or during sampling.

NO_3_^−^+NO_2_^−^ concentrations in waters ≤10 m were significantly lower (Mann–Whitney *U* test, *n*_May_ = 24, *n*_Oct_ = 28, *U* = 90, *p* < 0.01) during October (<0.03 µmol l^−1^) than in May (0.57 ± 1.10 µmol l^−1^) (Fig. 2C, H). The pattern was similar at depths between 11 and 50 m, with NO_3_^−^+NO_2_^−^ concentrations averaging 8.01 ± 7.10 µmol l^−1^ in May and 2.43 ± 3.65 µmol l^−1^ in October (Mann–Whitney U test, *n*_May_ = 30, *n*_Oct_ = 34, *U* = 257, *p* < 0.01). Below 50 m, where waters were influenced by Equatorial Subsurface water (Fig. S1), NO_3_^−^+NO_2_^−^ concentrations in May (20.22 ± 8.42 µmol l^−1^) and October (21.30 ± 8.29 µmol l^−1^) were similar (Mann–Whitney *U* test, *n*_May_ = 35, *n*_Oct_ = 47, *U* = 717, *p* > 0.05). Like NO_3_^−^, average surface water (≤10 m) PO_4_^3−^ concentrations were also higher in May (0.29 ± 0.79 µmol l^−1^) than in October (0.05 ± 0.02 µmol l^−1^) (Mann–Whitney *U* test, *n*_May_ = 24, *n*_Oct_ = 26, *U* = 81, *p* < 0.01, Fig. 2D, I) and no seasonal difference was detected below 50 m (May—1.28 ± 0.58 µmol l^−1^, October—1.44 ± 0.53 µmol l^−1^, Mann–Whitney *U* test, *n*_May_ = 35, *n*_Oct_ = 47, *U* = 647, *p* > 0.05). Both nitraclines and phosphoclines generally deepened with distance from the coast in May and along T3 in October, but varied little with depth across T1, T2, and T4 in October.

P* (P* = PO_4_^3−^ − (NO_3_^−^ + NO_2_^−^)/16), the amount of dissolved PO_4_^3−^ in the environment relative to what is expected if N and P uptake and remineralization proceed according to Redfield proportions [67], was on average slightly positive across the study region (Tables S1 and S2), suggestive of conditions conducive to N_2_ fixation, and possibly reflecting a signature of ODZ-derived waters that are transported into the Southern California Bight by the California Undercurrent [68]. In May, P* ranged from −0.81 to 3.91 µmol l^−1^, while in October the range was smaller (−0.20 to 0.52 µmol l^−1^). P* varied little with depth or distance from the coast.

At the surface (<10 m), May Chl *a* concentrations were higher than in October, 0.8 ± 1.17 vs. 0.1 ± 0.04 mg Chl *a* l^−1^ (Fig. 2E, J). Chl *a* concentrations were also higher in May at depths between 11 and 50 m (0.6 ± 1.22 vs. 0.3 ± 0.16 mg Chl *a* l^−1^), but deep Chl *a* maxima were more prominent in October, evidenced by the peak in Chl *a* concentration between 11–50 m (Figs. 2J and S1). Chl *a* concentrations generally decreased with distance from coast.

### Diazotroph community composition

**Fig. 3:**
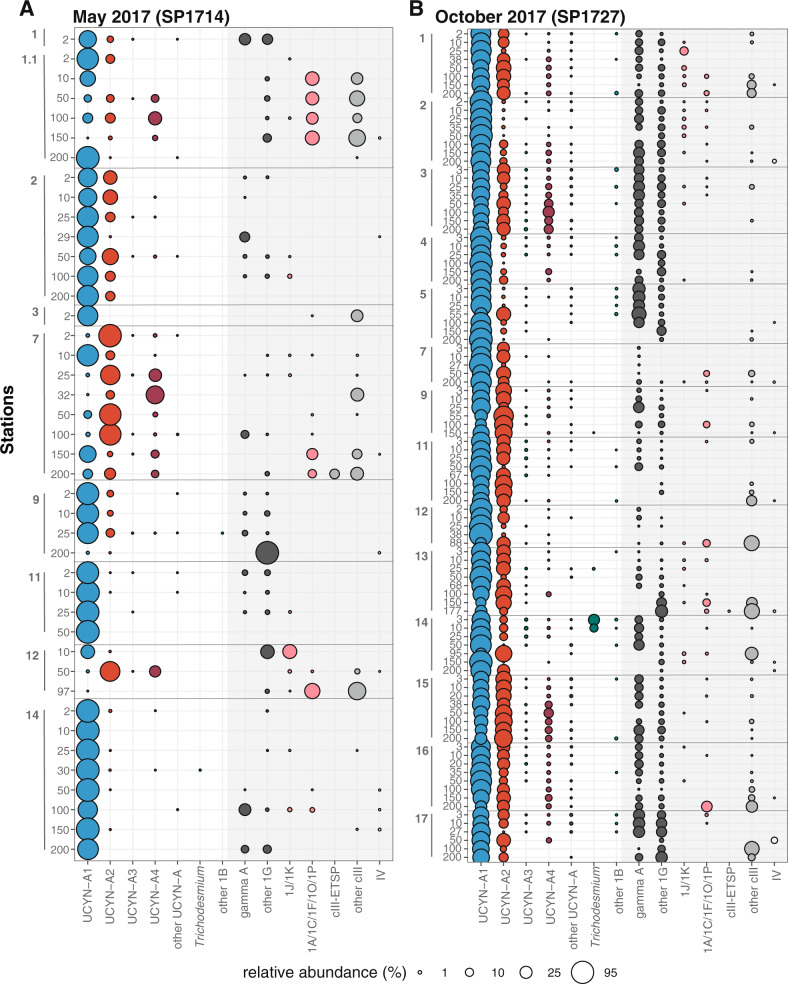
SCCS diazotroph community composition. Relative abundance of diazotrophs based on the sequencing of partial *nifH* genes during May 2017 (**A**) and October 2017 (**B**) cruises. Columns represent diazotroph taxonomic groups, and the size of the bubble scales to relative abundances for each group at each station/depth (rows). NCD taxonomic groups are identified using shaded columns, and gray lines delineate stations. Cluster designations are as described in Zehr et al. [45].

Partial *nifH* genes were amplified from all samples (76 from May and 105 from October); 148 remained after resampling (49 from May and 99 from October). The rarefied dataset is represented by 2178 OTUs. The majority of sequences affiliated with UCYN-A sublineages (82.6%), cluster 1G (putative γ-proteobacteria; 9.4%), and cluster III (putative δ- proteobacteria and other anaerobes; 4.8%) (Fig. 3). Relative abundances of the cyanobacterial diazotrophs *Trichodesmium*, *Crocosphaera*, *Cyanothece*, and *Richelia* associated with *Rhizosolenia* (Het-1) and *Hemiaulus* (Het-2) were low (0.2%).

Non-cyanobacterial diazotrophs (NCDs) accounted for 17.2% of the sequences, had high relative abundances in May, and were recovered from surface waters and below the deep chlorophyll max (Fig. 3A). Cluster 1G sequences were predominantly affiliated with gamma A [69] which had high relative abundances in October along T1 and T4 (Fig. 3B). Cluster III sequences were found in deeper samples (>50 m) and were dominated by several OTUs (Fig. 3). The most highly recovered cluster III OTU, denovo5, had 99% nucleotide identity to a South Pacific sequence type, cIII-ETSP [70]. The second most abundant cluster III OTU, denovo9, was not similar to any previously reported *nifH* sequence. Notably, two additional NCD OTUs have previously been reported: denovo13 which is similar to a *Klebsiella*-like OTU (OTU0009; [11]) and denovo30, which is identical to a Western Pacific sequence type, Alpha-MH144511 [71].

### UCYN-A *nifH* oligotyping

UCYN-A sublineages are comprised of multiple oligotypes but global sequence libraries are dominated by oligo1 (UCYN-A1), oligo2 (UCYN-A3), and oligo3 (UCYN-A2) [47]. SCCS UCYN-A assemblages contained 89 oligotypes;  34 were previously unreported but together accounted for less than 1% of UCYN-A sequences. The five oligotypes with the highest relative abundance were oligo1 (71.5%), oligo3 (20.7%), oligo4 (affiliated with UCYN-A4; 2.5%), oligo46 (UCYN-A2; 1.0%), and oligo45 (UCYN-A1; 0.5%) (Figs. S3 and S4). UCYN-A4 oligotypes (dominated by oligo4) had high relative abundances in some nearshore stations at depths >25 m (Figs. 3, S3, and S4), particularly May Stns. 1.1 and 7 and along the two northernmost transects in October (Figs. S3 and S4).

**Fig. 4:**
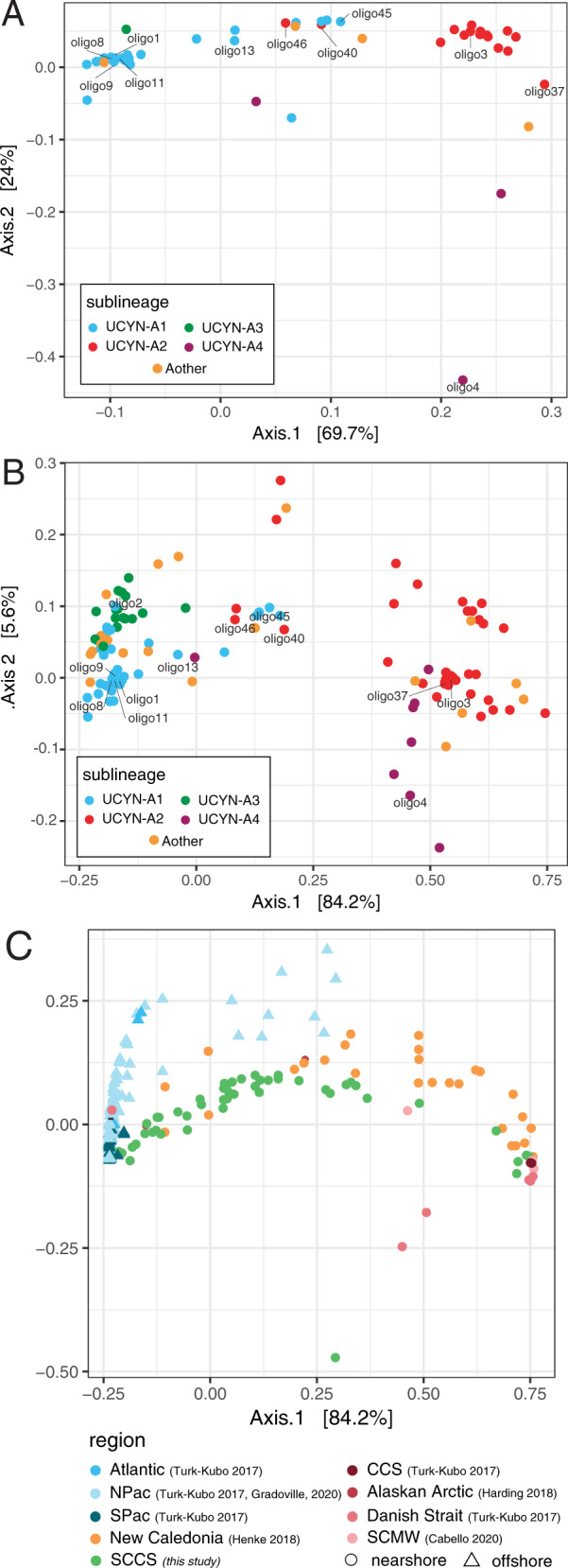
Patterns of UCYN-A *nifH* oligotype biogeography. Dissimilarity between samples is based on Principal Coordinate Analysis using the Bray–Curtis ecological index. Co-occurrence of SCCS UCYN-A oligotypes in the SCCS (**A**). Oligotypes with the highest relative abundances are labeled. Patterns in UCYN-A biogeography using samples collected in nearshore and offshore environments throughout the global oceans (**B**). Dissimilarity patterns for UCYN-A *nifH* oligotypes (**B**) and samples (**C**) indicate that SCCS samples fall between nearshore (circle) and offshore (triangle) endpoints, which are characterized by UCYN-A2 and UCYN-A1 symbioses, respectively. SCCS data were combined with other recent datasets [17, 37, 47–49].

PCoA using the Bray–Curtis dissimilarity index indicates that oligo1 co-occurred with minor UCYN-A1 oligotypes oligo8, oligo9, and oligo11, and most UCYN-A2 oligotypes co-occurred and cluster separately from UCYN-A1 (Fig. 4). Oligo4 co-occurred with UCYN-A2 along the PCoA axis 1. Notably, oligo46 (UCYN-A2) and oligo45 (UCYN-A1) formed a separate cluster (along with oligo13 and oligo40) that deviated from the predominant co-occurrence of oligotypes within a given sublineage. Oligo46 and oligo45 were found at Stn. 2 in May samples and throughout the region in October (Figs. S3 and S4).

### Abundance and distribution of targeted diazotrophs

**Fig. 5:**
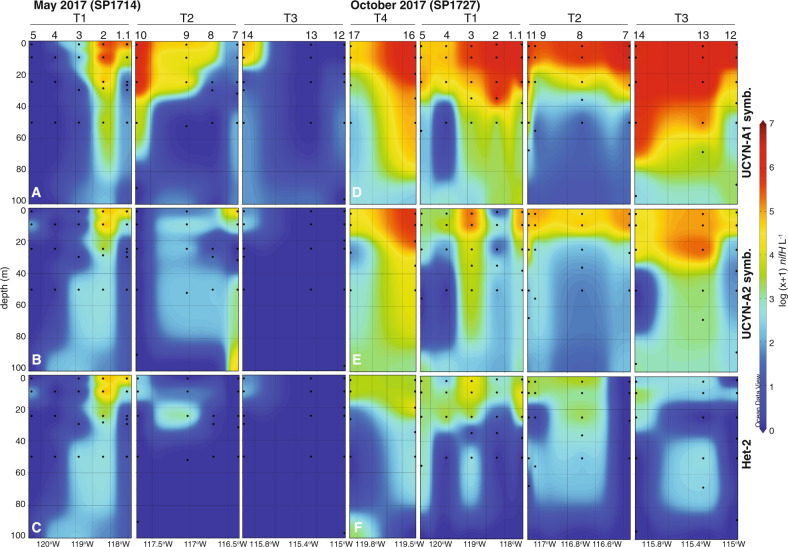
Patterns in qPCR-based abundances of dominant diazotroph taxa. Distributions of UCYN-A1 symbiosis (**A**, **D**), UCYN-A2 symbiosis (**B**, **E**), and Het-2 (**C**, **F**) in May 2017 (**A**–**C**) and October 2017 (**D**–**F**). Stations and transects are indicated at the top. Stn. 14 (October, T4) is not included due to missing surface data. Diazotrophs present at low abundances or sporadically detected are not included (Tables S1 and S2).

UCYN-A symbioses were the most abundant diazotrophs and detected in nearly all surface samples during both cruises. Average surface abundances of the UCYN-A1 symbiosis in October were significantly (Mann–Whitney *U* test, *n*_*May*_ = 8, *n*_*Oct*_ = 14, *U* = 20, *p* < 0.01) higher than in May, at 1.6 × 10^6^ ± 1.4 × 10^6^ and 3.6 × 10^5^ ± 4.7 × 10^5^ *nifH* l^−1^, respectively (Fig. 5, Tables S1 and S2), and in October, maximum abundances were generally associated with waters carrying a signature of tropical surface water (Fig. S1). Surface abundances of the UCYN-A2 symbiosis were lower and did not significantly differ between cruises, with May averages of 1.8 × 10^5^ ± 2.5 × 10^5^ *nifH* l^−1^ and October averages of 1.2 × 10^5^ ± 1.7 × 10^4^ *nifH* l^−1^. UCYN-A1 and UCYN-A2 symbiosis abundances were positively and significantly associated across both cruises (*ρ* = 0.71, *p* < 0.001). In addition, both had significant association with temperature and oxygen (*ρ* > 1) and depth, NO_3_^−^+NO_2_^−^, PO_4_^3-^ and σθ (*ρ* < 1; Table S3).

May UCYN-A symbioses distribution patterns were heterogenous. Peak UCYN-A1 symbiosis abundance along T1 was in warm, surface coastal waters above a shoaling nitracline, while found offshore along T2 and T3 (Figs. 2, 5A, S5a). UCYN-A2 symbiosis distribution patterns were similar, with the exception of T2, where high volumetric and depth-integrated abundances were measured at nearshore Stn. 7 (Figs. 5B and S5C).

In October, both symbioses were distributed throughout surface waters along all four transects and were detected at every station (Figs. 5D, E and S5B, D). Notably, the UCYN-A1 symbiosis was detected at high abundances in nearshore stations along T1–T3 (Fig. 5D). Considering only October data, UCYN-A1 symbiosis abundances were significantly and inversely (*ρ* < 1) associated with depth, salinity, NO_3_^−^+NO_2_^−^, PO_4_^3−^ and σθ, and positively (*ρ* > 1) associated with temperature, oxygen and Chl *a*. In addition, total UCYN-A symbioses abundance was significantly and inversely associated with P* during October (*ρ* = −0.21, *p* = 0.03) but not May (*ρ* = 0.14, *p* = 0.21; Table S3).

The second most abundant N_2_-fixer was Het-2, with peak abundances of 2.1 × 10^3^ ± 1.3 × 10^2^ *nifH* l^−1^ (Stn. 9, 25 m) and 2.2 × 10^4^ ± 6.8 × 10^2^ *nifH* l^−1^ (Stn. 16, 20 m) in May and October, respectively (Fig. 5C, F, Tables S1 and S2). In May, Het-2 was undetected or DNQ in most samples, except for Stn. 9. Depth-integrated abundances were highest in October, notably along the northern two transects (Fig. S5E, F). Het-2 abundance was significantly and positively associated with UCYN-A1 (*ρ* = 0.60, *p* < 0.001) and UCYN-A2 (*ρ* = 0.54, *p* < 0.001) symbioses abundances and temperature, oxygen and PAR, and significantly and inversely associated with depth, salinity, NO_3_^−^+NO_2_^−^, PO_4_^3−^, σθ (Table S3).

*Trichodesmium*, *Crocosphaera* (UCYN-B), and gamma A were sporadically detected (Tables S1 and S2). *Trichodesmium*, present at low abundance in October, had peak abundances at Stn. 14 (3.6 × 10^4^ ± 4.2 × 10^1^ *nifH* l^−1^). *Crocosphaera* was DNQ at two stations in May (Stns. 4, 10), and at three stations in October (Stns. 2, 7, 13) and only quantifiable in one October sample (Stn. 14, 50 m, 5.1 × 10^2^ ± 5.1 × 10^0^ *nifH* l^−1^). Gamma A was abundant in Stn. 1 surface waters in May (1.4 × 10^5^ ± 3.4 × 10^4^ *nifH* l^−1^) and at Stn. 9 (1.5 × 10^4^ ± 7.5 × 10^3^ *nifH* l^−1^) but was undetected in all October surface samples. Gamma A sequences were recovered from virtually all October samples (Fig. 3B), but qPCR data indicated they were below quantitation (<25 *nifH* l^−1^), consistent with known over-representation in sequence libraries [72].

### Bulk community N_2_ fixation rates

In May, N_2_ fixation was detected at the surface and deep chlorophyll maximum at every station (except Stn. 8 where NFR was not measured), but October NFRs were patchy. May NFRs averaged 8.5 ± 6.5 nmol N l^−1^d^−1^ in surface waters and 6.7 ± 3.3 nmol N l^−1^ d^−1^ at the deep chlorophyll maximum (Tables 1 and S4). The highest May volumetric rates were measured in nearshore surface waters at Stn 1.1 (14.2 ± 4.5 nmol N l^−1^ d^−1^) and Stn. 13 (23.0 ± 3.8 nmol N l^−1^ d^−1^). Concurrent with deeper MLDs and higher upwelling indices, N_2_ fixation was often detected at the deep chlorophyll maximum in the presence of NO_3_^−^+NO_2_^−^ concentrations >1 µM. May NFRs were not significantly associated with environmental parameters or diazotroph abundances (Table S3).

**Table 1:** Compilation of environmental parameters and volumetric euphotic NFR during May 2017 (SP1714) and October 2017 (SP1727).

Cruise	Station	Depth (m)	Temp. (°C)	Salinity	Nitrate + Nitrite (µM)	Phosphate (µM)	P*	Chl *a* (mg l^−1^)	NFR (nmol N l^−1^ d^−1^)	NFR LOD (nmol N l^−1^ d^−1^)
SP1714	1	2	18.22	33.40	0.05	0.09	0.08	0.173 ± 0.003	2.3 ± 1.3	0.4
	1	30^a^	11.91	33.41	9.82	0.88	0.27	0.900 ± 0.048	3.1 ± 0.7	0.4
	1.1	2	16.82	33.41	0.01	0.01	0.01	0.213 ± 0.004	14.2 ± 4.5	0.7
	1.1	27^a^	12.33	33.36	nm	nm	na	3.012 ± 0.046	11.5 ± 13.1	0.5
	2	2	16.32	33.42	0.10	0.07	0.07	0.508 ± 0.003	6.7 ± 0.7	0.3
	2	29^a^	13.63	33.27	9.05	0.64	0.07	0.950 ± 0.033	3.2 ± 2.0	0.6
	3	2	nm	nm	1.65	0.13	0.03	0.943 ± 0.009	4.8 ± 1.9	0.6
	3	25^a^	14.37	33.47	nm	nm	na	0.877 ± 0.013	4.0 ± 0.9	0.7
	4	2	nm	nm	nm	nm	na	nm	5.2 ± 1.4	0.9
	4	25^a^	14.18	33.32	1.31	0.19	0.11	0.509 ± 0.010	8.2 ± 6.4	1.0
	5	2	14.54	33.23	0.12	0.10	0.09	0.307 ± 0.013	7.1 ± 3.6	0.5
	5	50	14.51	33.23	0.28	0.06	0.05	0.283 ± 0.013	6.4 ± 2.9	0.5
	7	10	14.16	33.31	4.58	0.45	0.16	0.257 ± 0.006	6.1 ± 5.3	1.1
	7	32^a^	12.15	33.37	12.73	0.75	−0.04	0.275 ± 0.012	9.5 ± 1.6	0.8
	9	10	17.70	33.43	−0.01	0.05	0.05	0.164 ± 0.016	10.0 ± 2.6	0.5
	9	52^a^	13.53	33.26	4.38	0.32	0.05	0.398 ± 0.022	9.5 ± 2.6	0.3
	10	90	14.76	33.31	1.22	0.11	0.04	0.241 ± 0.006	3.2 ± 1.4	0.2
	12	2	16.10	33.51	0.02	0.06	0.06	0.765 ± 0.127	3.6 ± 2.0	0.4
	12	20^a^	13.47	33.44	4.97	0.59	0.28	1.884 ± 0.207	3.3 ± 0.9	0.6
	13	2	14.99	33.59	0.25	0.31	0.29	3.994 ± 0.055	23.0 ± 3.8	2.3
	13	10	13.60	33.49	2.84	0.33	0.15	4.268 ± 0.173	DNQ	2.5
	13	25^a^	13.54	33.58	4.60	0.30	0.02	6.044 ± 0.916	11.1 ± 7.4	3.0
	14	2	18.03	33.53	0.04	0.14	0.14	0.178 ± 0.009	9.9 ± 4.9	0.8
	14	10	18.00	33.52	0.02	0.16	0.16	0.174 ± 0.013	9.0 ± 4.1	0.7
	14	30^a^	14.09	33.18	4.55	0.43	0.15	1.133 ± 0.084	8.0 ± 7.9	1.4
SP1727	1	2	21.76	33.56	0.04	0.02	0.02	0.120 ± 0.007	2.2 ± 0.2	0.7
	1	10	21.35	33.51	0.00	0.02	0.02	0.149 ± 0.010	5.1 ± 0.2	1.8
	1	38^a^	14.41	33.32	2.22	0.27	0.13	0.595 ± 0.058	BDL	2.8
	2	2	20.46	33.58	0.00	0.03	0.03	0.115 ± 0.005	3.9 ± 0.2	1.4
	2	10	20.47	33.58	0.00	0.03	0.03	0.110 ± 0.001	5.8 ± 0.8	2.0
	2	35^a^	13.71	33.25	1.03	0.19	0.13	0.495 ± 0.017	BDL	3.3
	3	3	18.91	33.51	0.00	0.02	0.02	0.188 ± 0.013	5.7 ± 0.2	1.2
	3	10	18.88	33.49	0.00	0.07	0.07	0.201 ± 0.006	6.3 ± 0.4	1.5
	3	35^a^	14.62	33.16	0.21	0.11	0.10	0.390 ± 0.010	BDL	1.0
	4	3	18.58	33.44	0.00	0.07	0.07	0.100 ± 0.001	BDL	0.6
	4	10	18.58	33.44	0.00	0.08	0.08	0.095 ± 0.003	BDL	0.6
	4	50^a^	14.07	33.20	0.65	0.16	0.12	0.249 ± 0.002	BDL	0.5
	5	3	18.90	33.43	0.00	0.09	0.09	0.099 ± 0.001	BDL	1.0
	5	10	18.83	33.43	0.00	0.07	0.07	0.105 ± 0.005	BDL	0.7
	5	55^a^	14.26	33.34	0.24	0.13	0.12	0.309 ± 0.012	BDL	0.5
	7	3	19.95	33.39	0.00	0.07	0.07	0.161 ± 0.007	6.7 ± 0.8	1.2
	7	10	19.54	33.23	0.00	0.07	0.07	0.196 ± 0.010	16.5 ± 1.8	3.3
	7	27^a^	13.71	33.23	0.26	0.22	0.20	0.689 ± 0.044	BDL	5.4
	9	3	20.77	33.51	0.00	0.03	0.03	0.092 ± 0.012	DNQ	0.4
	9	10	20.75	33.50	0.00	0.03	0.03	0.094 ± 0.003	5.8 ± 1.2	0.9
	9	55^a^	13.85	33.27	1.46	0.23	0.14	0.271 ± 0.014	DNQ	0.8
	11	3	21.04	33.56	0.00	nm	na	0.088 ± 0.000	5.9 ± 0.9	0.6
	11	10	21.02	33.57	0.00	0.04	0.04	0.091 ± 0.003	5.8 ± 0.38	0.5
	11	67^a^	14.11	33.29	0.06	0.18	0.18	0.431 ± 0.015	BDL	0.5
	12	2	20.18	33.44	0.00	0.05	0.05	0.142 ± 0.003	14.1 ± 10.0	0.3
	12	10	19.76	33.34	0.00	0.05	0.05	0.154 ± 0.010	7.0 ± 0.2	0.6
	12	38^a^	14.80	33.29	1.73	0.33	0.22	0.678 ± 0.024	BDL	0.9
	13	3	21.14	33.50	0.00	0.04	0.04	0.098 ± 0.003	9.3 ± 1.9	0.5
	13	10	21.14	33.50	0.00	0.05	0.05	0.093 ± 0.001	19.6 ± 1.5	1.1
	13	68^a^	13.96	33.30	0.78	0.25	0.20	0.269 ± 0.011	1.4 ± 1.3	1.2
	14	3	21.74	33.57	0.00	0.05	0.05	0.063 ± 0.004	nm	nm
	14	10	21.53	33.56	0.00	0.05	0.05	0.078 ± 0.002	3.4 ± 0.2	1.1
	14	50	18.28	33.33	0.00	0.08	0.08	0.105 ± 0.005	BDL	1.6
	15	3	19.31	33.43	nm	nm	na	0.021 ± 0.006	7.3 ± 0.6	0.6
	15	10	19.26	33.41	nm	nm	na	0.211 ± 0.008	DNQ	0.8
	15	38^a^	14.05	33.30	nm	nm	na	0.868 ± 0.039	DNQ	0.6
	17	3	17.67	33.42	0.00	0.05	0.05	0.314 ± 0.129	BDL	1.5
	17	10	17.47	33.42	0.00	0.05	0.05	0.388 ± 0.037	BDL	1.9
	17	27^a^	15.95	33.28	1.40	0.18	0.09	1.494 ± 0.402	BDL	2.9

Detection limits and minimum quantifiable rates are detailed in Table S4.

*nm* not measured, *na* not applicable; *DNQ* detected not quantified, *BDL* below detection limit.

^a^Depth in the vicinity of the deep chlorophyll maximum.

NFRs were lower in October, with surface rates averaging 4.3 ± 4.4 nmol N l^−1^ d^−1^ and near or below detection limits at the deep chlorophyll maximum. NFRs in October were highest at 10 m at Stns. 13 (19.6 ± 1.5 nmol N l^−1^ d^−1^) and 7 (16.5 ± 1.8 nmol N l^−1^ d^−1^). October NFRs were significantly and positively associated with temperature, salinity, UCYN-A1 and UCYN-A2 symbioses abundance, and inversely associated with depth, fluorescence, oxygen, σθ, NO_3_^−^+NO_2_^−^, PO_4_^3−^, P* and Chl *a* (Table S3).

Depth-integrated NFR ranged from 62.0–409.3 µmol N m^−2^ d^−1^ in May (Fig. S5G), and from 0–709.1 µmol N m^−2^ d^−1^ in October (Fig. S5H). In May, high depth-integrated NFRs were measured in both nearshore and offshore stations, while in October, depth-integrated NFR was highest at Stn. 13, where the bottom depth was less than 200 m (Table S2, Fig. S5H).

### UCYN-A single-cell N_2_ fixation rates

**Fig. 6:**
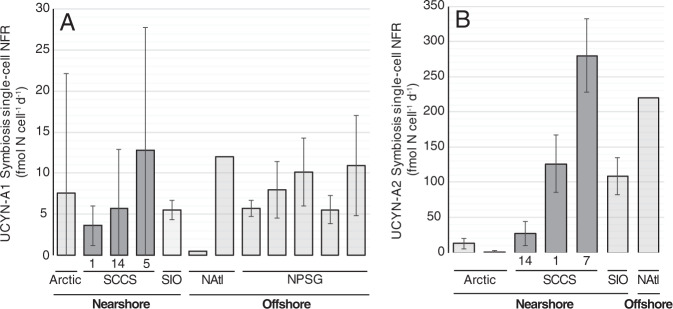
Compilation of UCYN-A symbioses single-cell N_2_ fixation rates. UCYN-A1 symbiosis (**A**) and UCYN-A2 symbiosis (**B**) single-cell NFRs in the SCCS are similar to rates reported in offshore waters. SCCS stations are identified on the *x*-axis. Note the different scales on the y-axis. Data compiled from the following studies where rates from intact symbioses are reported: “Arctic” [17], “SIO” [29], “Natl” [28], and “NPSG” [49].

Single-cell NFRs for the UCYN-A1 symbiosis were measured at Stn. 1 (May), Stn. 5 (October) and Stn. 14 (October), and ranged from BDL—30.5 fmol N cell^−1^ d^−1^, with an average of 6.6 ± 8.8 fmol N cell^−1^ d^−1^ (Fig. 6, Tables 2 and S5). Per cell N quotas ranged from 2.6–27.7 fmol N cell^−1^ (Table S5). UCYN-A1 symbiosis single-cell NFRs had high variability, most notably at Stn. 5 (October), where the average rate was 13.1 ± 14.6 fmol N cell^−1^ d^−1^, and below detection in 3 of 6 associations (Table S5).

**Table 2:** Estimated contribution of UCYN-A1 and UCYN-A2 symbioses to community NFRs at select near- and offshore stations.

Cruise	Stn	UCYN-A1/UCYN-A2	Number of cells	Measured			Estimated		Corrected estimate (maximum)		
				UCYN-A single-cell NFR (fmol N cell^−1^ d^−1^)	UCYN-A abundance (*nifH* copies l^−1^)	Community NFR (nmol N l^−1^ d^−1^)	UCYN-A volumetric NFR (nmol N l^−1^ d^−1^)	UCYN-A contribution to community NFR	Maximum UCYN-A single-cell NFR (fmol N cell^−1^ d^−1^)	Maximum UCYN-A volumetric NFR (nmol N l^−1^ d^−1^)	Maximum UCYN-A contribution to community NFR
SP1714	1	A1	10	3.6 (2.2)	4.1 × 10^4^ (7.3 × 10^2^)	2.3 (1.3)	0.15 (0.0)	6% (43%)	14.4	0.6	26%
SP1727	1	A2	6	125.8 (41.6)	9.3 × 10^4^ (4.2 × 10^3^)	2.2 (0.2)	1.7 (0.1)^c^	53% (16%)	503.2	4.7	213%
SP1727	5	A1	6	12.8 (13.5)	6.2 × 10^4^ (1.5 × 10^3^)	0.8 (0.1)^a^	0.8 (0.01)	99% (6%)	51.2	3.2	397%
SP1727	7	A2	6	279.9 (53.7)	1.2 × 10^5^ (2.9 × 10^3^)	6.7 (0.8)	3.6 (0.1)^c^	50% (28%)	1119.6	134	201%
SP1727	14	A1	10	5.7 (5.4)	8.8 × 10^5^ (8.1 × 10^4^)	3.4 (0.2)^b^	5.0 (2.7)	148% (270%)	22.8	20.1	590%
SP1727	14	A2	5	27.0 (15.4)	2.9 × 10^4^ (1.1 × 10^2^)	3.4 (0.2)^b^	0.8 (0.0)^c^	2% (7%)	108	0.3	9%

Standard deviations for measured parameters and propagated errors for estimated parameters are in parenthesis. Maximum single-cell NFR using correction factors defined by [64], and the resulting maximum UCYN-A volumetric NFR and contribution to community are also reported.

^a^ NFR was BDL, value used is the detection limit.

^b^ NFR data from 10 m; N content at 3 m was below levels needed for accurate and/or precise isotopic measurement.

^c^ Potential contributions to volumetric NFR rates for the UCYN-A2 symbiosis assume that there are 10 *nifH* copies cell^−1^ [64].

Single-cell NFRs for the UCYN-A2 symbiosis were measured at three stations in October (Stns. 1, 7, and 14) and ranged from 2.2–362.2 fmol N cell^−1^ d^−1^, with an average of 151.1 ± 112.7 fmol N cell^−1^ d^−1^. Per cell N quotas ranged from 49.3–159.8 fmol N cell^−1^ (Table S5). UCYN-A2 symbiosis single-cell NFR was the highest nearshore at Stn. 7 (279.9 ± 52.1 fmol N cell^−1^ d^−1^) and lowest offshore at Stn. 14 (27.0 ± 16.7 fmol N cell^−1^ d^−1^).

## Discussion

### N_2_ fixation in the SCCS

Marine N cycle measurements have not been focused on N_2_ fixation in coastal SCCS waters; however, our study demonstrates that it is widespread along the continental shelf between the Southern California Bight and Sebastián Vizcaíno Bay, and in adjacent offshore waters. NFRs in surface waters were high throughout the study region in both May and October and volumetric rates fall into the mid-to-high range of previously reported rates in nearshore environments (Table S6). Together with rates measured in the northern portion of the Southern California Bight, the Eastern Tropical North Pacific, and the Gulf of California [8, 73, 74], our study extends the region of N_2_ fixation in the SCCS. The relationships between chemical and physical properties of the water column and bulk N_2_ fixation are broadly consistent with the general paradigm of marine N_2_ fixation occurring in warm, sunlit, N-deplete ocean waters [75], particularly during the fall oceanic season, where NFRs were positively associated with temperature and inversely associated with depth, NO_3_^−^+NO_2_^−^, and Chl *a* (Tables S3, S7, and S8). However, whole community NFRs in this region appear insensitive to excess PO_4_^3−^ (P*), which may reflect the dominance of UCYN-A and the insensitivity of N_2_ fixation by the UCYN-A symbioses to nitrate concentrations [29, 63].

Despite this overall trend, some of the highest NFRs were measured in May, when upwelling conditions existed 4 days prior to the measurements (Fig. S2). The upwelling was reflected in lower sea surface temperatures and higher NO_3_^−^+NO_2_^−^, PO_4_^3−^, and Chl *a* concentrations, compared to October. Notably, one station was occupied before (Stn. 1 on May 3, 2017) and after (Stn 1.1 on May 9, 2017) the upwelling event and surface NFRs significantly were higher after the upwelling, at 2.3 ± 1.3 prior to and 14.2 ± 4.5 post-upwelling (unpaired *t*-test, *p* = 0.012). At this station, UCYN-A1 and UCYN-A2 symbioses abundance increased by 1 and 2 orders of magnitude post-upwelling, respectively, and Chl *a* concentrations  from the surface to 50 m also increased (Tables 1 and S1). Together these observations suggest a link between post-upwelling conditions and increased NFRs by UCYN-A symbioses. However, a focused field campaign designed to test this directly is needed to reproduce these results and determine whether post-upwelling conditions differentially stimulate NFRs in UCYN-A1 vs. UCYN-A2 symbioses.

Enhanced primary productivity is suspected to stimulate N_2_ fixation in UCYN-A symbioses. A link between UCYN-A symbioses presence and/or activity and indicators of primary productivity (namely Chl *a*) has been reported [6, 24], but the underlying mechanism(s) remain unidentified. However, stimulation of NFRs in the UCYN-A1 symbiosis upon the addition of NO_3_^−^, despite the lack of NO_3_^−^ assimilation by the haptophyte host, has been experimentally demonstrated and is speculated to result from changes in the productivity or activity of the broader microbial community [29]. The production of organic matter, vitamins (e.g., B_12_), or Fe-binding compounds by bacterioplankton and phytoplankton is well documented [76–78], and in environments such as the SCCS, which experience upwelling-stimulated net primary production, may be particularly important to the productivity of UCYN-A symbioses.

Averaged across the study region, depth-integrated NFR was markedly similar between the two study periods (May—195 ± 108 µmol N m^−2^ d^−1^, October—195 ± 189 µmol N m^−2^ d^−1^). Assuming Redfield C:N proportions for phytoplankton, this N would support the production of ca. 1.4 mmol C m^−2^ d^−1^. The climatological estimate (1970–2008) of new production in our study region was determined to be 17 and 1.7 mmol C m^−2^ d^−1^ in May and October, respectively [79]. Thus, at the time of this study, N_2_ fixation accounted for ~8% of new production in May and 83% in October. The climatological estimate for total production in the region was greater and not substantially different between May (37.5 mmol C m^−2^ d^−1^) and October (27.5 mmol C m^−2^ d^−1^) [79]. As such, N_2_ fixation-based production would only contribute to ~4–5% of total production. Future estimates of new production for the region are lower due to community composition shifts (less diatoms and more picophytoplankton) [80]. Such future changes would likely increase the role of N_2_ fixation in supporting new production. Improved representation in ecological models of the magnitude and seasonality of nearshore N_2_ fixation as well as the activity of coastal diazotrophs in the SCCS and other coastal regions will aid our understanding of and ability to predict changes to the balance between the global ocean’s N sources (N_2_ fixation) and sinks (denitrification and anammox), particularly in coastally influenced waters rapidly undergoing climatological changes.

### Contribution of UCYN-A symbioses to SCCS N_2_ fixation

Our study indicates that UCYN-A symbioses are important N_2_-fixers in the SCCS. This is supported by high UCYN-A abundances and low abundances of other diazotrophs, along with the highest single-cell NFR rates reported (UCYN-A2 symbiosis; 362 fmol N cell^−1^ d^−1^, Table S5), and average rates comparable to those reported from offshore regions (Fig. 6). Furthermore, UCYN-A symbioses can account for a substantial portion, if not all, of the bulk N_2_ fixation at each station, with the exception of Stn. 1 (sampled May 3, prior to the upwelling event). Although our estimates are limited to a subset of stations and subject to large errors, N_2_ fixation by the UCYN-A symbioses can account for between 6% and up to 100% of bulk N_2_ fixation (Table 2). A substantial percentage of bulk N_2_ fixation measured at offshore stations is attributed to UCYN-A1 symbioses, while the UCYN-A2 symbiosis appears to contribute predominantly to nearshore waters, which is a new line of evidence supporting the hypothesis that sublineages are different ecotypes [47]. This is most evident at Stn. 14 where potential contributions from the UCYN-A2 symbiosis were low (2 ± 7%) even though the UCYN-A1 symbiosis could account for bulk NFRs.

Estimates of the UCYN-A symbioses contribution to bulk rates are valuable but require cautious interpretation. The accuracy of cell abundance estimates using qPCR is impacted by numerous factors including DNA extraction efficiency, the quality of qPCR standards, and qPCR assay specificity/efficiency, and can be complicated by polyploidy, which is not well known in UCYN-A. Detecting ^15^N assimilation into single cells using nanoSIMS is subject to large uncertainties resulting from error associated with each measured parameter (e.g., $A_{\it{PN}}^{\it{final}}$, $A_{\it{PN}}^{t = 0}$), the small number of data points, and underestimates of ^15^N incorporation due to isotope dilution [64]. More direct measurements of UCYN-A single-cell NFRs in other temperate coastal regions are needed, but our findings confirm their importance to N_2_ fixation in coastal ecosystems.

It should be noted that heterocyst-forming *Richelia* associated with diatoms (diatom/diazotroph associations; DDAs) may contribute to nearshore N_2_ fixation. They have been reported in the SCCS [29, 81] and Gulf of California [73, 82], and were detected at low abundances throughout the region in October, However, DDAs have some of the highest per cell NFRs reported [83, 84], thus, even at low abundances they have the potential to be substantive contributors to bulk N_2_ fixation [85].

### UCYN-A symbioses assemblages reflect mixing of nearshore and offshore ecotypes

SCCS UCYN-A assemblages were dominated by the UCYN-A1 symbiosis, even in nearshore stations, which was surprising given it was undetected in a 2010–2011 seasonal study in the Santa Catalina Bight [52]. Regional warming trends have been hypothesized to increase advection of oligotrophic waters into the Southern California Bight [68] and SST remained elevated in 2017 after the anomalous 2014–2016 warming trend and the 2015 El Niño (0.5–1 °C SST anomalies between 28–32° N; [86]), which have been linked to the persistence of warm *Prochlorococcus* ecotypes into 2018 [87] and may also explain the high abundances of the UCYN-A1 symbiosis in 2017.

In addition, UCYN-A oligotype assemblages reflect a mixture of nearshore and offshore populations in the SCCS. Ordination analysis on the global ocean dataset shows offshore samples clustered together and apart from nearshore samples (Fig. 4C) driven by the co-occurrence of UCYN-A1/UCYN-A3 in offshore samples and UCYN-A2/UCYN-A4 in nearshore samples (Fig. 4B) and are consistent with previously described patterns [47]. SCCS and Noumea Lagoon samples were found in each cluster, reflecting sample-specific similarities to both offshore and nearshore assemblages.

However, several oligotypes were found primarily in coastal waters influenced by the advection of oligotrophic waters. Many SCCS and Noumea Lagoon samples did not cluster with offshore and nearshore endpoints, and these dissimilarities are influenced by a mixture of UCYN-A1 (oligo45) and UCYN-A2 oligotypes (oligo46 and oligo40), along with oligo4 (Fig. 4B). Morphological and genetic studies of *B. bigelowii* suggest it is a complex of biologically distinct species, many of which can be found in coastal environments [88]. Thus, minor oligotypes such as oligo45, 46, and 40 may be affiliated with *B. bigelowii* genotypes distinct from known hosts. However, it is unclear whether all *B. bigelowii* genotypes form symbioses with UCYN-A and more work is needed to link oligotypes with genotypes.

### Summary and conclusions

This work directly demonstrates that UCYN-A symbioses actively fix N_2_ in the temperate, coastal SCCS ecosystem, where their cell-specific rates are high enough to account for whole community N_2_ fixation. N_2_ fixation by UCYN-A1 was more important in offshore waters, while UCYN-A2 contributed a higher share of the total N_2_ fixation in nearshore waters, suggesting that even when these sublineages co-occur, their activity may be influenced by different environmental factors. More measurements in other coastal ecosystems are needed to better understand the variability and environmental controls on UCYN-A N_2_ fixation, however, these findings lend support to speculation about its importance to N_2_ fixation in other coastal systems [6, 7, 10, 89, 90]. In addition, although whole community N_2_ fixation does not account for a large amount of new production in this system, this work establishes that N_2_ fixation is a widespread process along the Baja California peninsula, during both upwelling and oceanic seasons and throughout the euphotic zone, with the highest volumetric rates in euphotic waters overlying the continental shelf. These measurements provide an important baseline for understanding how N_2_ fixation and N-cycling will be impacted by future changes in climatology of the SCCS.

## Supplementary information

Supplementary Material
