# Supplementary material for: UCYN-A/haptophyte symbioses dominate N2 fixation in the Southern California Current System
Source: ISME Commun. 2021 Aug 26;1:42. doi: 10.1038/s43705-021-00039-7 (PMC9723760; doi:10.1038/s43705-021-00039-7)
Supplement: Supplementary file 1 — Supplementary Material [file 43705_2021_39_MOESM1_ESM.docx]

**Supplemental Methods**

*General linear models*

General linear models were used to evaluate the relationships between environmental (predictor) variables and UCYN-A/haptophyte symbioses abundances, as well as predictor variables (including N_2_-fixer abundances) and N_2_ fixation rates (NFRs). A set of composite factors were defined using a Principle Component Analysis (PCA), which accounts for the multicollinearity of the environmental parameters. Factor loadings were rotated using varimax rotation, which best aligns the original predictor variables on individual principle components (PC). Imputation was used, allowing calculation of PC scores for the few samples that had missing values for predictor variables. Only PC’s with eigenvalues >1 were retained and used in the general linear models (1, 2).

In one iteration, the PCA components (Factor 1, Factor 2, Factor 3, and Factor 4) were considered predictor variables and UCYNA1 log(*nifH* L^-1^+1) and UCYN-A2 log(*nifH* L^-1^+1) were used as response variables in two separate models. Reduced models were determined by sequentially dropping PCA components not contributing to model fit using Akaike information criterion (AIC) standards. Variance inflation factor (VIF) scores for continuous variables were much less than 10 indicating the lack of co-linearity at a level that would compromise the modeling (3). These models provide the relationship (positive/negative) and statistical significance between environmental variables and UCYNA1 log(*nifH* L^-1^+1) and UCYN-A2 log(*nifH* L^-1^+1).

In the second iteration, log(*nifH* L^-1^+1) of all targeted diazotrophs were added as predictor variables (except gammaA) and NFR (nmol N L^-1^ d^-1^) was used as the response variable. NFRs that were below detection limits (BDL) were set to 0, and below minimum quantifiable rates (MQR) were set to the MQR (Table S6). These analyses utilized JMP®, Version Pro 12 (SAS Institute Inc., Cary, NC).

**Supplemental Results**

*Environmental determinants of UCYN-A1 and UCYN-A2 abundance and NFR*

In order to determine which environmental factors can be used to predict SCCS UCYN-A/haptophyte symbiosis abundance, reduced models were developed with composite factors from a PCA as the independent variables and UCYN-A1 and UCYN-A2 log (x+1) *nifH* L^-1^ as the dependent variables. Models were run on the entire dataset (SP1714+SP1727) and on each cruise separately (Table S7). Reduced models for UCYN-A1 and UCYN-A2 for the entire dataset were significant (adjusted R^2^ of 0.31 and 0.17, respectively) and explanatory (p<0.0001). The UCYN-A1 model had two predictor variables (Factor 1 and Factor 3) both of which were significant (p<0.0001) in each model. The UCYN-A2 model had three significant predictor variables, Factor 1 (p<0.0001), Factor 2 (p = 0.0868) and Factor 3 (p = 0.006). Factor 1 included depth, salinity, σθ, NO3-+NO2-, PO32-., PAR, temperature, and oxygen. Factor 2 included latitude and longitude. Factor 3 included Chl *a*, CTD fluorescence, and bottom depth.

The negative sign of the parameter estimates for Factor 1 in both the UCYN-A1 and the UCYN-A2 models indicated a negative relationship between Factor 1 and UCYN-A abundances after other parameters have been modeled. After taking into account which parameters have positive and negative factor loading in the PCA, the model suggests that as temperature, PAR and oxygen increase, UCYN-A abundance increases (direct relationship, “+” in Table S7), and as depth, salinity, σθ, NO_3_^-^+NO_2_^-^, and PO_4_^3-^ increase, UCYN-A abundance decrease (indirect relationship, “-“ in Table S7). Likewise, the negative sign of the parameter estimates for Factor 3 in both models suggests that as Chl *a* and CTD fluorescence increase, UCYN-A abundances decrease, and as bottom depth increases, UCYN-A abundances increase. The significance of Factor 2 in the UCYN-A2 model suggests that location, namely higher latitudes and lower longitude (relative to the overall study region) will have higher UCYN-A2 symbiosis abundances. Despite this small difference, collectively, these models indicate that the abundances of both UCYN-A symbioses are driven by similar environmental variables in the SCCS.

Reduced UCYN-A1 and UCYN-A2 models using only the SP1714 cruise dataset had lower adj. R^2^ (0.09 and 0.03, respectively) and were less explanatory (p = 0.0022 and p = 0.0477, respectively). Reduced UCYN-A1 and UCYN-A2 models using the SP1727 cruise dataset were more similar to the entire dataset (Table S7). A notable exception between the full and cruise-specific datasets is the inclusion of P* in the UCYN-A2 SP1714 and UCYN-A1 SP1727 model.

The reduced model for the entire dataset, developed with NFR as the dependent variable and composite PCA factors, including *nifH*-based abundances from qPCR, as the independent variables, had an adjusted R^2^ of 0.21 and was explanatory (p=0.0004) based on two predictor variables, Factor 3, which included bottom depth, CTD fluorescence and Chl *a*, Factor 3, which included latitude and longitude, and Factor 5, which included PAR, P*, UCYN-B *nifH* L^-1^, and *Trichodesmium* *nifH* L^-1^ (Table S8). Accounting for the sign of the parameter estimate and the loadings on the PCA, the model suggests that NFR increases with increasing latitude and decreases with increasing PAR, P*, and the abundances of UCYN-B and *Trichodesmium*.

The reduced NFR model could not be developed using only SP1714 data, but the SP1727 model had an adjusted R^2^ of 0.50 and was explanatory (p<0.0001) based on two predictor variables, Factor 1, which included depth, temperature, salinity, oxygen, σθ, NO_3_^-^+NO_2_^-^, UCYN-A1 *nifH* L^-1^, UCYN-A2 *nifH* L^-1^ and Het-2, *nifH* L^-1^ and Factor 3, which included latitude, longitude and P* (Table S8). Accounting for the sign of the parameter estimate and the loadings on the PCA, the model suggests that October NFR increases with increasing temperature, oxygen, longitude, P*, UCYN-A1 *nifH* L^-1^, UCYN-A2 *nifH* L^-1^ and Het-2, *nifH* L^-1^and decreases with increasing depth, salinity, sq, NO_3_^-^+NO_2_^-^, and latitude.

**Supplemental Tables**

Table S1. Hydrological, biogeochemical and N_2_-fixer qPCR-derived abundance data for May 2017 (SP1714) SCCS cruise. '--' parameter not measured; DNQ - detected, not quantified; UD - undetected.

Table S2. Hydrological, biogeochemical and N_2_-fixer qPCR-derived abundance data for October 2017 (SP1727) SCCS cruise. '--' parameter not measured; DNQ - detected, not quantified; UD - undetected.

Table S3. Spearman’s rank coefficients (ρ) and significance between diazotroph abundances, NFR and environmental variables. Analyses were conducted on the entire dataset, and on each cruise separately. Significant (p < 0.05) associations are emphasized in bold.

Table S4. Whole community NFR. LOD and MQR are defined according to (4).

Table S5. Single-cell NFR (SC-NFR). LOD and MQR are defined according to (4).

Table S6. Compiled volumetric and depth integrated NFR from euphotic nearshore environments. See references in Supplemental File. BDL = below detection limits; “--” – not reported; SCCS - Southern California Current System; CCS - California Current System; ETNP - Eastern Tropical North Pacific; OMZ - Oxygen minimum zone; NP - North Pacific.

|  | Region | NFR range (nmolN L^-1^d^-1^) | Depth Integrated NFR range (µmolN m^-2^d^-1^) | Reference |
| --- | --- | --- | --- | --- |
| Eastern North Pacific | SCCS | BDL - 23 | 0 - 709 | this study |
|  | CCS (offshore) | 0.18 - 0.24 | 10 | (5) |
|  | Gulf of California | BDL - 24 | 20-250 | (6) |
|  | Southern California Bight | 0.3 - 36 | 760 | (7) |
|  | Gulf of California/ETNP | BDL - 31 | 795 | (8) |
|  | ETNP OMZ | BDL - 4 | -- | (9) |
|  | Coastal ETNP | BDL - 89 | 14 - 6226 | (10) |
| Western North Pacific | Northwestern NP | BDL - 13.6 | 0 - 294 | (11) |
| Western Atlantic | Georges Bank | BDL - 9 | 0 - 1911 | (12) |
|  | Gulf of Maine | BDL - 37 | 0 - 514 | (12) |
|  | Mid Atlantic Bight | BLD - 89 | 0 - 1694 | (12, 13) |
|  | Southern New England Shelf | BLD - 130 | 0 - 4106 | (12) |
|  | Mid Atlantic Bight | -- | 3000 | (14) |
| Eastern Atlantic | Iberian Peninsula | 0.001 - 0.095 | -- | (15) |
|  | Benguela Upwelling System | 8-Feb | 85 | (16) |
|  | English Channel | 18 - 20 | 350 | (17) |
| Danish Strait | Roskilde Fjord | BDL - 47 | -- | (18) |
|  | Great Belt | BDL - 83 | -- | (18) |
| South Pacific | New Caledonian Lagoon | -- | Sep-87 | (19) |
|  | New Caledonian Lagoon | 0.6 - 1.3 | -- | (20) |
|  | South China Sea | 0 - 29 | 2 -191 | (21) |
|  | South China Sea (Mekong River Plume) | 0.11-22.77 | -- | (22) |
|  | Kuroshio and East China Sea | 0 - 28 | 232 | (23) |
|  | Taiwan Strait | BDL - 7.51 | -- | (24) |

Table S7. Linear Models to assess the relationship between predictor variables and *nifH* L^-1^; Effect Test with Parameter Estimates. P-values (Prob > F) indicate the fit of individual terms. The sign of parameter estimates indicates if the predictor variable is directly related (+) or inversely related (-) to PCA factors. Linear models developed for the entire dataset (SP1714+SP1727) and for each cruise separately.

| Dataset | Model | Source | DF | Sum of Squares | F Ratio | Prob > F | Parameter Estimates |
| --- | --- | --- | --- | --- | --- | --- | --- |
| SP1714 + SP1727 | UCYN-A1 symbiosis | Factor 1 | 1 | 198.1 | 69.1 | <0.0001 | -1.0 |
|  |  | *(-) depth, (-) salinity, (-) sq, (-) N+N, (-) phosp., (+) PAR, (+) temp, (+) oxygen* | | | | | |
|  |  | Factor 3 | 1 | 53.4 | 18.6 | <0.0001 | -0.6 |
|  |  | *(-) chl a, (-) CTD fluorescence, (+) bottom depth* | | | | | |
|  | UCYN-A2 symbiosis | Factor 1 | 1 | 68.9 | 26.7 | <0.0001 | -0.6 |
|  |  | *(-) depth, (-) salinity, (-) sq, (-) N+N, (-) phosp., (+) PAR, (+) temp, (+) oxygen* | | | | | |
|  |  | Factor 2 | 1 | 7.6 | 3.0 | 0.0868 | -0.2 |
|  |  | *(+) lat, (-) long* | | | | | |
|  |  | Factor 3 | 1 | 31.5 | 12.2 | 0.0006 | -0.5 |
|  |  | *(-) chl a, (-) CTD fluorescence, (+) bottom depth* | | | | | |
| SP1714 | UCYN-A1 symbiosis | Factor 1 | 1 | 29.9 | 10 | 0.0022 | -0.6 |
|  |  | *(-) depth, (-) salinity, (-) sq, (-) N+N, (-) phosp., (+) temp, (+) oxygen* | | | | | |
|  | UCYN-A2 symbiosis | Factor 3 | 1 | 9.1 | 4 | 0.0477 | 0.31 |
|  |  | *(+) lat, (-) long, (+) P** | | | | | |
| SP1727 | UCYN-A1 symbiosis | Factor 1 | 1 | 143.7 | 139.9 | <0.0001 | -1.1 |
|  |  | *(-) depth, (-) salinity, (-) sq, (-) N+N, (-) phosp., (+) temp, (+) oxygen* | | | | | |
|  |  | Factor 2 | 1 | 13.9 | 13.6 | 0.0004 | -0.4 |
|  |  | *(-) chl a, (-) CTD fluorescence, (+) bottom depth, (+) PAR* | | | | | |
|  |  | Factor 3 | 1 | 5.8 | 5.6 | 0.0197 | 0.2 |
|  |  | *(-) lat, (+) long, (+) P** | | | | | |
|  | UCYN-A2 symbiosis | Factor 1 | 1 | 43.2 | 26.3 | <0.0001 | -0.6 |
|  |  | *(-) depth, (-) salinity, (-) sq, (-) N+N, (-) phosp., (+) temp, (+) oxygen* | | | | | |
|  |  | Factor 2 | 1 | 9.9 | 6 | 0.0157 | -0.3 |
|  |  | *(-) chl a, (-) CTD fluorescence, (+) bottom depth, (+) PAR* | | | | | |

Table S8. Linear Models to assess the relationship between predictor variables and NFR – Effect Test with Parameter Estimates. P-values (Prob > F) indicate the fit of individual terms. The sign of parameter estimates indicates if the predictor variable is directly related (+) or inversely related (-) to PCA factors. Linear models developed for the entire dataset (SP1714+SP1727) and for SP1727.

| Dataset | Model | Source | DF | Sum of Squares | F Ratio | Prob > F | Parameter Estimates |
| --- | --- | --- | --- | --- | --- | --- | --- |
| SP1714 + SP1727 | NFR | Factor 3 | 1 | 11.9 | 15.3 | 0.0002 | 0.4 |
|  |  | *(-) latitude, (+) longitude* | | | | | |
|  |  | Factor5 | 1 | 4.2 | 5.4 | 0.0232 | -0.4 |
|  |  | *(-) PAR, (-) P*, (-) UCYN-B* nifH *L^-1^, (-) Tricho* nifH *L^-1^* | | | | | |
| SP1727 | NFR | Factor 1 | 1 | 16.1 | 31 | <0.0001 | -0.9 |
|  |  | *(-) depth, (+) temp., (-) salinity, (+) oxygen, (-) st, (-) N+N, (+) UCYN-A1* nifH *L^-1^, (+) UCYN-A2* nifH *L^-1^, (+) Het-2* nifH *L^-1^* | | | | | |
|  |  | Factor 3 | 1 | 3.4 | 6.6 | 0.0148 | 0.3 |
|  |  | *(-) latitude, (+) longitude, (+) P** | | | | | |

**Supplemental Figures**

Fig. S1 was S5. θ-S diagrams for SP1714 and SP1727 cruises. Derived potential temperature (θ) was calculated from CTD temperature and salinity data. Depth, nutrients, chl a, and diazotroph abundances are plotted in θ-S space in the smaller panels for each cruise and labeled accordingly. StSW – Subtropical surface water; SAW – Subarctic Water; ESsW – Equatorial Subsurface Water; TrW – Transitional water; NPIW – North Pacific Intermediate Water; PDW – Pacific Deep Water.

Fig. S2 was S1. Bakun upwelling indices in the week prior to and throughout SP1714 (A) and SP1727 (B) cruises. T1 is represented by 33N 119W, T2 by 30N 119W, and T3 by 27N 116W.

Fig. S3 was S2. UCYN-A *nifH* oligotype relative abundances in May 2017. Major oligotypes (A), minor oligotypes (B).


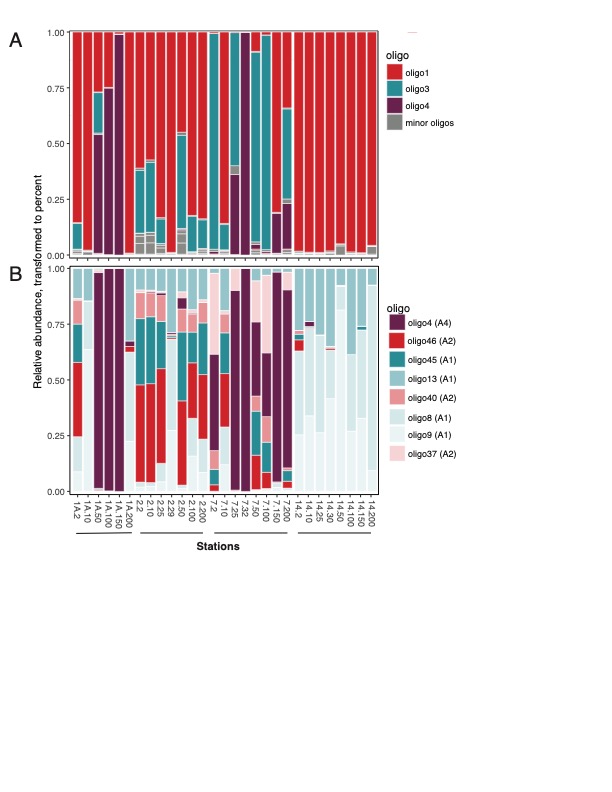


Fig. S4 was S3. UCYN-A *nifH* oligotype relative abundances in October 2017. Major oligotypes (A), minor oligotypes (B).


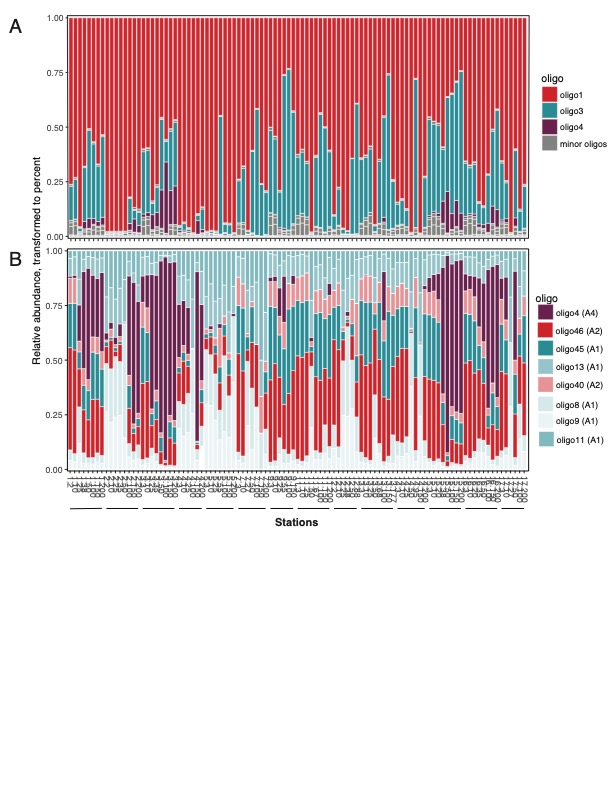

Fig. S5 was S4. Depth integrated *nifH*-based abundances and NFR the southern California Coastal System. Depth integrated UCNA-A1 abundances from May 2017 (A) and October 2017 (B). Depth integrated UCNA-A2 abundances from May 2017 (C) and October 2017 (D). Depth integrated Het-2 abundances from May 2017 (E) and October 2017 (F). Depth integrated NFR based on volumetric rate measurements from between 2-3 depths in the photic zone in May 2017 (G) and October 2017 (H). NFR rates were not measured at Stn. 8 (both cruises) or Stn. 16 (October) (*).

**Supplemental References**

1. Zar JH. Biostatistical Analysis, 4th edn. . Upper Saddle River, NJ, USA: Prentice Hall; 1999.

2. Quinn GP, Keough MJ. Experimental Design and Data Analysis for Biologists. Cambridge, UK.: Cambridge University Press; 2002.

3. Zuur AF, Ieno EN, Elphick CS. A protocol for data exploration to avoid common statistical problems. *Methods Ecol. Evol*. **1**:3-14 (2010).

4. Gradoville MR, *et al.;* Diversity and activity of nitrogen‐fixing communities across ocean basins. *Limnol. Oceanogr.* **62**:1895-1909 (2017).

5. Needoba JA, Foster RA, Sakamoto C, Zehr JP, Johnson KS. Nitrogen fixation by unicellular diazotrophic cyanobacteria in the temperate oligotrophic North Pacific Ocean. *Limnol. Oceanogr.* **52**:1317-1327 (2007).

6. White AE, Prahl FG, Letelier RM, Popp BN. Summer surface waters in the Gulf of California: Prime habitat for biological N_2_ fixation. *Global Biogeochem. Cy.* **21**, GB2017 (2007).

7. Hamersley MR *et al.;* Nitrogen fixation within the water column associated with two hypoxic basins in the Southern California Bight. *Aquat. Microb. Ecol.* **63**, 193-205 (2011).

8. White AE, *et al.;* Nitrogen fixation in the Gulf of California and the Eastern Tropical North Pacific. *Prog Oceanogr.* **109**, 1-17 (2013).

9. Jayakumar A, *et al.;* Biological nitrogen fixation in the oxygen-minimum region of the eastern tropical North Pacific ocean. *ISME J.* **11**, 2356-2367 (2017).

10. Selden CR, *et al.;* Dinitrogen Fixation Across Physico‐Chemical Gradients of the Eastern Tropical North Pacific Oxygen Deficient Zone. *Global Biogeochem. Cy.* **33**, 1187-202 (2019).

11. Shiozaki T, Nagata T, Ijichi M, Furuya K. Nitrogen fixation and the diazotroph community in the temperate coastal region of the northwestern North Pacific. *Biogeosciences* **12**, 4751-4764 (2015).

12. Mulholland MR, *et al.;* Rates of dinitrogen fixation and the abundance of diazotrophs in North American coastal waters between Cape Hatteras and Georges Bank. *Limnol. Oceanogr.* **57**:1067-1083 (2012).

13. Mulholland MR, *et al.;* High rates of N_2_ fixation in temperate, western North Atlantic coastal waters expands the realm of marine diazotrophy. *Global Biogeochem. Cy*. **33**, 826-840 (2019).

14. Tang W, *et al.;* Revisiting the distribution of oceanic N_2_ fixation and estimating diazotrophic contribution to marine production. *Nat. Commun.* **10**, 1-10 (2019).

15. Moreira-Coello V, *et al.;* Biological N_2_ Fixation in the Upwelling Region off NW Iberia: Magnitude, Relevance, and Players. *Front. Mar. Sci.* **4**, 303 (2017).

16. Sohm JA, *et al.;* Nitrogen fixation in the South Atlantic Gyre and the Benguela Upwelling system. *Geophys. Res. Lett.* **38**, L16608 (2011).

17. Rees AP, Gilbert JA, Kelly-Gerreyn BA. Nitrogen fixation in the western English Channel (NE Atlantic Ocean). *Mar. Ecol. Prog. Ser.* **374**, 7-12 (2009).

18. Bentzon-Tilia M, *et al.;* Significant N_2_ fixation by heterotrophs, photoheterotrophs and heterocystous cyanobacteria in two temperate estuaries. *ISME J.* **9**:273-285 (2015).

19. Bonnet S, *et al.;* Dynamics of N_2_ fixation and fate of diazotroph-derived nitrogen in a low-nutrient, low-chlorophyll ecosystem: results from the VAHINE mesocosm experiment (New Caledonia). *Biogeosciences* **13**, 2653-2673 (2016).

20. Benavides M, *et al.;* Dissolved organic matter influences N_2_ fixation in the New Caledonian lagoon (Western Tropical South Pacific). *Front. Mar. Sci*. **5**, 89 (2018).

21. Voss M, Bombar D, Loick N, Dippner JW. Riverine influence on nitrogen fixation in the upwelling region off Vietnam, South China Sea. *Geophys. Res. Lett*. **33**, L07604 (2006)

22. Grosse J, Bombar D, Doan HN, Nguyen LN, Voss M. The Mekong River plume fuels nitrogen fixation and determines phytoplankton species distribution in the South China Sea during low- and high-discharge season. *Limnol. Oceanogr.* **55**, 1668-80 (2010).

23. Shiozaki T, *et al.;* New estimation of N_2_ fixation in the western and central Pacific Ocean and its marginal seas. *Global Biogeochem. Cy.* **24**, GB1015 (2010).

24. Wen Z, *et al.;* Nitrogen fixation in two coastal upwelling regions of the Taiwan Strait. *Sci. Rep.* **7**, 1-10 (2017).

25. Meyer NR, Fortney J, Dekas AE. NanoSIMS sample preparation decreases isotope enrichment: magnitude, variability and implications for single-cell rates of microbial activity. *Environ. Microbiol.* **23**, 81-98 (2020).

26. Thompson A, *et al*. Genetic diversity of the unicellular nitrogen-fixing cyanobacteria UCYN-A and its prymnesiophyte host. *Environ. Microbiol.* **16**, 3238-3249 (2014).

27. Durazo R. Seasonality of the transitional region of the California Current System off Baja California. *J. Geophys. Res. Oceans* **120**, 1173-1196 (2015).
